# The complement system and kidney cancer: pathogenesis to clinical applications

**DOI:** 10.1172/JCI188351

**Published:** 2025-05-01

**Authors:** Ravikumar Aalinkeel, Richard J. Quigg, Jessy Alexander

**Affiliations:** Department of Medicine, University at Buffalo, Buffalo, New York, USA.

## Abstract

Kidney cancer poses unique clinical challenges because of its resistance to conventional treatments and its tendency to metastasize. The kidney is particularly susceptible to dysfunction of the complement system, an immune network that tumors often exploit. Recent discoveries have highlighted that the complement system not only plays a crucial role in immune surveillance and defense in the circulatory system, but also functions intracellularly and autonomously. This concept has shifted the focus of investigation toward understanding how complement proteins influence cancer progression by regulating the tumor microenvironment (TME), cell signaling, proliferation, metabolism, and the immune response. With the complement system and its inhibitors emerging as a promising new class of immunotherapeutics and potential complement-targeted treatments advancing through development pipelines and clinical trials, this Review provides a timely examination of how harnessing the complement system could lead to effective tumor treatments and how to strategically combine complement inhibitors with other cancer treatments, offering renewed hope in the fight against kidney cancer.

## Introduction

A critical aspect of cancer is the complement system, which often recognizes cancer cells as foreign entities and plays a role in their elimination (1). Inflammation influences tumor development across all stages, with the tumor microenvironment (TME) shaped by the interplay of infiltrating leukocytes and the complement system. The TME, cancer type, expression of complement-regulatory proteins, and interactions with other immune cells profoundly shape the role of complement in cancer progression, immune modulation, and metastasis. Renal cell carcinoma (RCC) presents unique clinical challenges due to its resistance to conventional therapies, high propensity for metastasis, and grim prognosis. As complement-based therapies continue to expand, it is imperative to enhance our understanding of the complement system’s role in kidney cancer and to identify potential biomarkers for clinical use. This manuscript aims to explore how advancements in understanding complement biology may revolutionize the diagnosis, treatment, and management of kidney cancer. Clarifying the role of complement in RCC may pave the way to identification of novel biomarkers and therapeutic targets with the potential to improve disease monitoring and advance personalized therapeutic strategies.

## The complement system

The complement system, which plays a critical role in host defense against pathogens, cancer, and damaged self-antigens (2, 3), operates through a tightly regulated enzymatic cascade, involving three main pathways: the classical, alternative, and lectin pathways. The cascade consists of approximately 50 soluble, membrane-bound, and intracellular proteins (4, 5). These pathways converge to form the convertases, leading to the assembly of the membrane attack complex (MAC) or C5b-9, which forms pores in the plasma membrane to lyse target cells. Each pathway is activated by different triggers: the classical pathway by the recognition of antigen-antibody complexes by C1q, the lectin pathway by pattern recognition molecules such as mannose-binding lectins, ficolins, and collectins binding to carbohydrates or glycoproteins on antigen surfaces (6), and the alternative pathway is continuously active, albeit at a low level.

Complement activation produces C3- and C5-convertases that cleave C3 into C3a and C3b, and C5 into C5a and C5b, respectively. C5b subsequently interacts with C6, C7, C8, and C9 to form the MAC, which causes osmotic lysis of target cells. Notably, sublytic MAC levels (7) and C5aR inhibition (8) can modulate VEGF, a key factor in tumor development, progression, and metastasis. Complement activation generates various effector molecules such as C2b, C3b, C4b, C5b, C4d, iC3b, C3dg, C3d, C2a, C4a, C3a, and C5a. C3b, C4b, C2b, and C5b participate in the formation of convertases, while C3a and C5a bind to their receptors (C3aR and C5aR) to recruit immune cells and regulate vascular permeability, proliferation, histamine release, and reactive oxygen species production. Additionally, C3a/C3aR interacts with CD46 to influence Th1 cell differentiation and T cell dynamics. Intracellular C3a, generated by the protease cathepsin L (CTSL) (9), is vital for T cell survival through mTOR activation. C5a/C5aR1 signaling promotes inflammasome assembly, IL-1β secretion, and Th1 activation (10).

**Figure 1:**
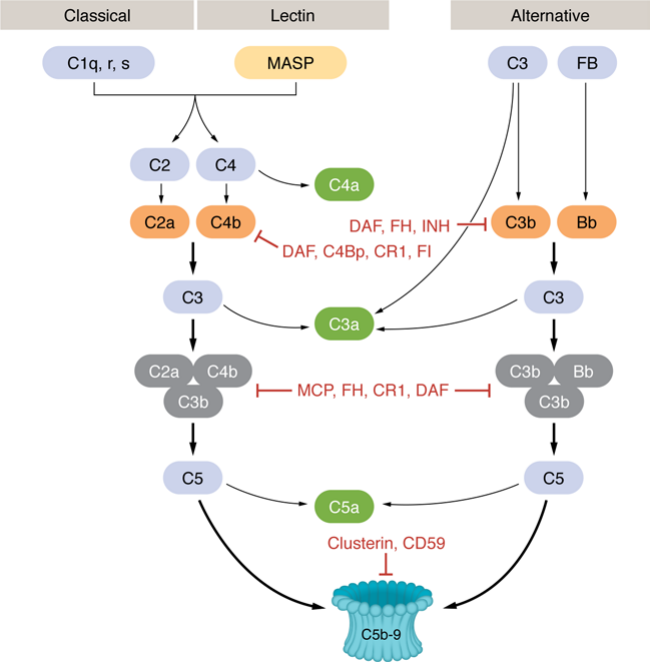
The complement cascade. The classical, lectin, and alternative pathways of the complement cascade converge on the assembly of C5b-9 MAC. C3 convertases appear in orange, C5 convertases appear in gray, and regulators of complement are indicated in red text. INH is the C1 inhibitor that regulates the classical, lectin, and alternative pathways.

Complement activation products can potentially damage self-tissues (Figure 1); therefore, C3a and C5a are removed by plasma carboxypeptidases, while C3b and C4b are deactivated by serine proteases (11). The complement system is tightly regulated by CR1, factor H (FH), CD46, CD55, and CD59, preventing inappropriate complement activation in fluid phases and cell surfaces. Complement proteins are ubiquitous (10, 12–16). However, the expression of complement proteins varies by location (17) and serves as a bridge between the innate and adaptive immune systems (18), enabling a more comprehensive and effective immune response.

The complement system also engages in noncanonical roles, including synaptic pruning (19) and autophagy (20). Consequently, deficiencies or dysfunctions in complement proteins are associated with numerous pathologies, including cancer (21–25). In the context of kidney cancer, the complement system exhibits a dual role: it enhances immune surveillance against tumors while potentially supporting tumor progression through mechanisms of immune evasion and inflammation.

## Tumor, TME, and complement

Tumors can develop in any part of the body, and their growth and response to treatment are influenced by a variety of factors, including environmental factors and lifestyle that shape the microbiome. The microbiome impacts (26–29) tumors by modulating Wnt signaling, p53 activity, and complement function (30). Additionally, the TME consists of a diverse, dynamic array of cells, including B cells, T cells, tumor-associated macrophages (TAMs), neutrophils (TANs), myeloid-derived suppressor cells (MDSCs), Tregs, DCs, fibroblasts, pericytes, and adipocytes, and creates a supportive environment for tumor growth, survival, proliferation, and metastasis (31–34).

The complement system in the TME enters through the tumor’s extensive vascular network or is produced by incoming immune cells or the tumors themselves (35) and can be triggered by tumor neoantigens (36). Both extracellular and intracellular (35) complement activity play important roles in cancer. For instance, tumor-derived C3a can activate TAMs through the C3a-C3aR/PI3K signaling pathway, which subsequently suppresses T cell function (37). C3a-C3aR signaling recruits neutrophils (38), promotes generation of neutrophil extracellular traps, and causes N2 polarization, thereby enhancing tumorigenesis. In TME, the complement system serves as a vital mediator, facilitating interactions that both promote and inhibit cancer progression, thereby influencing therapeutic responses and outcomes in kidney cancer.

### Complement and tumor suppression.

The complement system suppresses tumors through complement-dependent cytotoxicity (CDC) and tumor cell lysis (39–41). C5a influences the recruitment and differentiation of mesenchymal stem cells (MSCs), which in turn, secrete immunomodulators and checkpoint molecules (42–44), contributing to the immune landscape within the TME. In addition to the TME, complement proteins and receptors directly affect tumor cells. Preclinical and clinical observations show that complement activation correlates with T cell exhaustion/dysfunction and alternatively activated macrophages and regulates immunosuppression in human RCC. CD8^+^ T cell function is enhanced by reducing tumor growth in C3aR1KO mice, indicating that C3aR1 and C5aR1 regulate cytolytic activity of tumor-infiltrating lymphocytes (TILs). C5a also promotes the differentiation of CD4^+^ T cells into Tregs, further influencing the immune response (45). CD8^+^ and CD4^+^ T cells are activated and recruited by CXCL-10, which is secreted by a recently identified subpopulation of C1q^+^ TAMs within the TME. Additionally, pentraxins (PTX3) in the TME bind to C1q, functioning as tumor suppressors by modulating complement activation, carcinogenesis, inflammation, and angiogenesis (46). However, as inflammation persists and becomes chronic, the balance between tumor development and suppression shifts, resulting in a TME that increasingly supports tumor growth.

### *Complement and tumor survival*.

Conversely, the complement system supports tumor survival by several mechanisms (47, 48). The tumor survival mechanisms include evasion of CDC by upregulating soluble as well as membrane-bound complement regulators and receptors by tumor and stem cells (49–51); hindering the formation and stability of MAC by heat shock protein-90 (HSP-90) and mortalin (40, 52); activation of cancer-associated fibroblasts (CAFs) through C3a/C3aR signaling, promoting epithelial-mesenchymal transition (EMT) and metastasis, seen in breast cancer models (53); enhancement of ovarian cancer cell proliferation (54) and tumor progression through IL-1β/IL-17A signaling (55) and the PI3K/AKT pathway in an autocrine manner by C3aR and C5aR1 agonists (35); enhancement of tumor growth in orthotopic mouse tumor models, by C5a depleting CD8^+^ cytotoxic T cells and increasing the infiltration of MDSCs into the TME (56–59); depletion of C3 and C5aR1 inhibiting ovarian tumor growth regardless of the immune profile (8); promoting melanoma expansion by C3, produced by infiltrating CD8^+^ T cells inhibiting IL-10 production in an autocrine manner (60); C1q per se promoting tumor growth and metastasis (61); C1q/gC1qR signaling enhancing tumor proliferation by suppressing CD4^+^ T cell activity, similar to programmed cell death protein 1 (PD1) (62, 63); and lastly, sublytic MAC deposition on cancer cells, which fosters cell proliferation and resistance to apoptosis (64). This broad collection of tumor-suppressive and -promoting functions of complement underscores the need for a nuanced understanding of complement’s involvement in cancer, highlighting the potential for complement-based therapies and emphasizing the need for careful consideration of potential unintended consequences.

Tumor growth relies heavily on nutrient availability, anabolic processes, and waste removal, making angiogenesis a crucial factor (65). The complement system is integral to this process (47, 66), with anaphylatoxin signaling contributing to the recruitment of neutrophils and TAMs to inflammatory sites, while also modulating macrophage differentiation toward an M2-like phenotype that supports immunosuppression and tumor promotion (67, 68). Furthermore, complement activation in the TME can polarize neutrophils to a protumorigenic phenotype (N2-TAN) that produces proangiogenic factors, thereby increasing nutrient and oxygen availability and suppressing antitumor adaptive response by producing inducible NOS–inhibited (iNOS-inhibited) T cell activation (69–71). Resistance to anti-VEGF therapies has emerged as a profound challenge in cancer treatment, manifesting in several ways including adaptive resistance, rebound growth, and MDSC recruitment (72, 73). A combinatorial approach of anti-VEGF and complement therapeutics synergistically combat tumor growth by not only restricting tumor blood supply but also by unleashing a more potent antitumor immune response (74). Overall, the complement system supports angiogenesis by influencing VEGF expression and endothelial cell activity as well as by regulating stromal cells in the TME, thereby supporting tumor survival and growth (75).

## Kidney, complement, and cancer

The kidney contains nephrons that filter 20%–22% of the cardiac output, producing 1–2 liters of urine daily, and maintain fluid balance (76, 77). Glomeruli, the filtering units of the kidney, comprise specialized epithelial cells — the podocytes, mesodermal-derived mesangial cells that are crucial for tissue fibrosis, endothelial cells, which serve as the interface between circulating blood and the kidney, and fibroblasts that are located in the renal interstitium and contribute to the structural integrity of the kidney. (78, 79). The kidney tubules consist of proximal and distal convoluted tubule cells, collecting duct cells, loop of Henle cells, and principal and intercalated cells (80). These cells regulate electrolyte balance, fluid volume, and pH through filtration, reabsorption, and secretion. Their functions are influenced by their specific locations within the kidney and the surrounding microenvironment.

**Figure 2:**
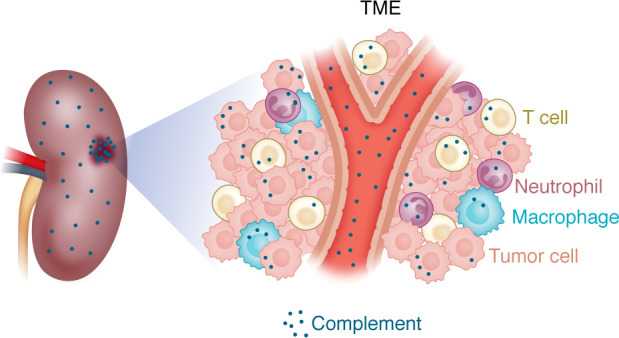
TME in kidney. Kidney cancer TME is dynamic with hematopoietic cells and complement. Complement can invade the TME through the blood or can be synthesized locally by the infiltrating immune cells, resident kidney cells, and tumor cells.

A range of complement proteins and complement regulators are synthesized locally in the kidneys (Figure 2) (81–84). A comprehensive review by Zhou et al. (85) details varying regional expression of complement proteins in the kidney, such as C2, C4, C3, and factor B being more prevalent in the renal cortex, while C1q and factor D are primarily found in the glomeruli (85). Regulators such as decay-accelerating factor (DAF/CD55), membrane cofactor protein (MCP/CD46), complement receptor type 1 (CR1/CD35), and MAC inhibition factor (MACIF/CD59) are also differentially expressed in the kidney. Notably, kidney tubules lack DAF and CD59, while the glomerular basement membrane lacks complement regulators, making them particularly vulnerable to complement-mediated damage. Within the kidney, a lineup of intrinsic positive and negative controls guards the renal structures through their impact on complement activation. C1q and CR1 in humans and FH in mice (86) play key roles in clearing immune complexes and apoptotic bodies. Local expression of proteins such as C1q and FH could have a kinetic advantage over deposition of a circulating component. The C5a receptor, C5aR1, is expressed on proximal tubular cells in the renal cortex, where it mediates inflammation and tubular injury in response to complement activation; the second C5a receptor, C5L2, is also expressed on renal tubular epithelial and interstitial cells, but is less prevalent compared with C5aR1 and appears to play a regulatory role, possibly attenuating C5aR1 signaling under certain conditions. Thus, the proteins are suggested to play contrasting roles modulating tumor evasion and survival (87, 88); however, the precise role of complement proteins in maintaining local kidney homeostasis remains incompletely understood (89).

### Kidney cancer.

Kidney cancers with distinct origins and histological characteristics accounts for about 5% of all malignancies and are associated with advancing age (90, 91), and, in the US, cancer incidence is projected to rise among adults aged 40–60 years (92). RCC ranges from stages I to IV, with approximately one-third of RCC patients presenting metastatic disease affecting other organs such as lungs at diagnosis (93). RCC are resistant to radio- and chemotherapy (94, 95), and about one-fifth of RCC patients will have a relapse and develop metastasis after nephrectomy (96). Although there have been substantial advances in therapies for RCC in recent years, each approach comes with its limitations. Surgical interventions could lead to reduced renal function and complications (97), and VEGF and mTOR inhibitors can lead to resistance and may cause side effects such as altered metabolism, hypertension, and fatigue (98). Not all patients respond to immunotherapies and patients may have adverse immune-related events, while combination therapies have increased toxicity compared to monotherapies (97, 98). Therefore, understanding tumor heterogeneity and the underlying molecular mechanisms is crucial for improving treatment outcomes. In RCC, dysregulation of complement activation within the TME plays a critical role in tumor progression and therapeutic response. RCC is often characterized by high expression of complement pathway proteins and is associated with a poor prognosis, leading to its classification as an “aggressive complement tumor” (99). Preclinical studies have explored the role of the complement system as a predictor of immune sensitivity in metastatic RCC (100, 101).

## Challenges in kidney cancer

Kidney cancers differ from nonrenal cancers in that the overexpression of complement-regulatory proteins in the kidney influences immune evasion and angiogenesis, while nonrenal cancers show a broader diversity in complement activation outcomes promoting immune activation (in some cancers) or inflammatory responses (in others). Kidney cancer susceptibility is influenced by genetic predispositions, lifestyle variations, and medications. One of the lifestyle links among the kidney, complement system, and cancer is the microbiome, which produces various necessary compounds such as the short-chain fatty acids (SCFAs) (102, 103), modulates processes including energy metabolism and immune responses (104), and protects the kidney against oxidative stress, mitochondrial dysfunction, and inflammation (105, 106). RCCs typically arise from the lining of kidney tubules. Damage or dysfunction in these tubules can lead to tubular acidosis, electrolyte imbalances, and compromised renal function. Cancers employ survival mechanisms to evade immune surveillance and therapeutic interventions, including mimicking viral infections that activate interferon pathways, resembling immune cells, and overexpressing or acquiring complement regulators from the local environment to shield themselves from immune detection (107, 108). Additionally, cancers may develop resistance mechanisms against complement-based therapies.

The complexity of cancer pathogenesis varies substantially based on the organ of origin, and even within the same organ, such as tumors located in different parts of the colon, which can differ in their mutations and histological features (109). Tumors can also show heterogeneity within themselves due to genetic variations among cells or changes over time. This inter- and intratumoral variability is made more complex by the multifaceted nature of the complement system. Similarly to tumors, complement components also differ in their localization, concentration, and response to the microenvironment. Research on C3-deficient kidneys has highlighted the critical role of locally generated complement in renal injury (110, 111). Anaphylatoxins C3a and C5a attract immune cells to sites of injury and inflammation, but C5a/C5aR signaling can also enhance IL-6 expression, which is associated with cell proliferation and cancer progression, including in RCC (112, 113).

Complement regulatory proteins, CD55, CD59, MCP, CD46, FH, and FH-like proteins are often upregulated in cancers (114) with variations among cancer types and tumor specimens of the same cancer type (115). Histochemical examination of RCC revealed that FH was expressed both as membrane bound and intracellular. Intracellular FH and not membrane FH was associated with worse outcome (116). C1q exhibits both pro- and antitumorigenic properties, potentially influencing tumor progression based on cancer type, location, receptor interactions, and the duration of complement signaling (61, 117). Rituximab, an anti-CD20 monoclonal antibody, activates the classical complement pathway both in vitro and in vivo (118, 119), with enhanced antitumor effects in complement-deficient mice (120). Conversely, eculizumab, a humanized anti-C5 antibody, is effective in some conditions (121), but less so in others with ongoing complement activation (122). This variability can be attributed to the complement system’s feedback loops and functional redundancies, which make predicting the impact of interventions challenging. However, its application to solid tumors such as kidney cancer has not been studied. C5 could play a dual role: promoting immune suppression through C5a-mediated recruitment of MDSCs and enabling tumor cell lysis through MAC formation. C5aR1 and C5aR2 (123) are present on kidney cancer cells (Human Protein Atlas v23, proteinatlas.org), especially clear cell RCC (ccRCC) cells, which originate from proximal tubules and play contrasting roles modulating tumor evasion and survival. C5aR1 is shown as a potential therapeutic target modulating actin rearrangement and thereby metastasis (124), while the studies on C5aR2 in cancer have yielded conflicting results, highlighting its complex role in various malignancies. In lung and breast cancer, C5AR2 together with IL-10 were robustly associated with chemoresistance (125); conversely, in a melanoma-bearing murine model, C5AR2 appeared to restrict tumor growth (126), and absence of C5AR2 increased tumor progression in azoxymethane/dextran sodium sulfate–induced (AOM/DSS-induced) colorectal cancer (CRC) tumorigenesis (a model of colitis-associated cancer), indicating that C5AR2 has an antiinflammatory effect (127). However, studies on C5aR2 in RCC are limited and further investigation is needed. Furthermore, complement system assessments often reflect only a specific moment in time, lacking a comprehensive view of the cascade’s effects. The integrity of patient samples during collection can also impact results (128). Understanding the mechanisms by which complement influences disease settings is crucial, as complement inhibition can lead to unintended consequences, such as increased susceptibility to infections, autoimmune conditions, and disruption of physiological functions.

Kidney cancer, particularly RCC, often remains asymptomatic for long periods, making early diagnosis challenging. Despite the rise in small, incidentally detected tumors through medical imaging, over 33% of patients are still undiagnosed at an advanced stage, indicating an urgent need for more effective diagnostic markers. The likelihood of recurrence in RCC can be as high as 40% in patients who have had surgery for a tumor that is still localized. Despite advancements in imaging technologies that have improved prognosis, RCC continues to have the highest mortality rate among all urological cancers. To better manage this challenging disease, it is crucial to predict its progression, response to systemic therapies, and to identify effective biomarkers for early detection. Developing robust prognostic models (129, 130) will enable more accurate, individualized survival predictions, leading to more tailored treatment plans. Metastatic RCC often necessitates risk stratification to guide targeted therapies. Identifying definitive prognostic factors and creating multivariate models will enhance the ability to predict disease progression and recurrence, thereby improving postsurgery monitoring and potentially integrating biomarkers to refine current prognostic algorithms. Moreover, since the impact of complement proteins varies based on their location within the body, effective targeting of complement in metastatic RCC and other cancers may require different methods of administration to access various locations.

## Prognostic potential of complement proteins in RCC

Complement proteins have emerged as promising prognostic markers in RCC. A series of studies examined complement activation in RCC and its impact on clinical outcomes using original tumor samples from three distinct cohorts (comprising 106, 154, and 43 individuals), RCC cell lines, and tumor models in complement-deficient mice (116, 131, 132). The key findings include the following: (a) elevated levels of classical pathway components (C1q, C4, C4 activation fragments) and C4d deposits are associated with poor prognosis in RCC (116, 131, 132); (b) intracellular and not extracellular C1s correlate with increased macrophages and T cells, independent of complement deposits. C1s inhibition reduced cell growth and survival, suggesting protumoral activities independent of the complement cascade (132, 133); (c) tumoral C5a and not C3aR serves as an independent negative predictor of postsurgery outcomes in a study of limited patients by IHC and scoring, particularly in advanced stages and high-risk groups and correlate with adverse clinical outcomes in RCC (134); (d) C3 and FN1 upregulation in RCC tissues correlates with lower overall survival rates (135); (e) PTX3 triggers C1q and releases proangiogenic factors such as C5a, and its upregulation in RCC is associated with poorer survival rates (136); (f) assessment of 272 ccRCC patients revealed that elevated tumoral C5a is associated with poor overall survival and could significantly stratify patient prognosis in advanced and intermediate/high risk group, and incorporating other parameters improved the predicting accuracy (137, 138); and (g) overexpression of 11 complement genes correlates with unfavorable prognosis (100), T cell dysfunction markers, and alternatively activated macrophages in RCC, with complement proteins primarily expressed or deposited in the tumor stroma, leukocytes, and vasculature, except for CD59, which is associated with favorable outcomes. In addition, this study developed an algorithm able to stratify patients by high or low likelihood of responding to immune checkpoint inhibitors (ICIs) based on their plasma measurements of MAC and C5a.

Complement-based markers have yet to be implemented in clinical practice. Although the studies described provide valuable insights, several limitations and outstanding questions need to be addressed. The lag in implementing complement markers in the clinic may stem from (a) lack of clarity on which specific markers in the complement cascade are most effective with respect to their sensitivity, specificity, and applicability as potential prognostic markers to the different RCC subtypes and long-term outcomes; (b) difficulties in identifying RCC patients who would benefit from complement-based therapies; (c) the need to establish the impact of sample size on result reliability and generalizability, since studies reviewed here are of small and variable sample sizes (43 to 272 individuals); (d) strong correlations in the studies but no definite causality, e.g., CD59’s favorable prognosis compared with other complement components, which warrants further validation; (e) the interaction with immune checkpoints and the synergy between complement therapies and immunotherapies; and (f) the systemic effects of targeting complement in RCC patients. Additionally, the relationship between complement components and cancer varies depending on the disease stage. Although limitations and questions highlight the need for further research to develop effective complement-prognostic markers, these studies suggest that incorporating complement components as biomarkers, alongside other prognostic parameters, could enhance the precision of RCC prognosis.

## Complement system as therapeutic targets

Current research is exploring various therapeutic targets for RCC, including anti-CTLA4 antibodies (139), angiogenesis inhibitors (140), tyrosine kinase inhibitors, extracellular matrix inhibitors, and myeloid cell targets (141). Given the strong association between complement components and poor prognosis in RCC, targeting the complement system is emerging as a promising approach. However, treatment decisions largely depend on baseline clinical and laboratory parameters and the stage of the cancer, with initial strategies focusing on tumor resection and surgery. ICIs (142) are currently leading in therapeutic options, but the landscape is rapidly evolving with new potential targets. Although combination therapies have improved clinical outcomes, some patients do not respond to first-line treatments, and certain combination regimens can still be toxic. This underscores the urgent need for alternative therapies and biomarkers to predict treatment response.

Recent advances in drug discovery have introduced a growing list of specific and effective complement inhibitors. There are over 850 clinical trials and 190 drug candidates, with 11 FDA-approved complement medicines showing promise for RCC and other complement-related diseases. Complement therapeutics offer several potential benefits: they can serve as adjuvants (143) to disrupt tumor evasion mechanisms, are compatible with traditional chemotherapies without affecting common immunosuppressive pathways, generally have fewer side effects, and can be delivered via various routes. Moreover, complement inhibitors can be effectively combined with ICIs due to their distinct modes of action. While checkpoint inhibitors boost cytotoxic T cell proliferation, complement inhibitors reduce the infiltration of MDSCs into the TME, thereby mitigating MDSC-induced T cell suppression and enhancing T cell function. However, using anticomplement therapies requires careful consideration, as they might interfere with the effectiveness of other antitumor agents. For instance, combining anticomplement agents with monoclonal antibodies like cetuximab or rituximab could reduce the antibodies’ effectiveness, especially if CDC is crucial. Adding anti-CD59 can alleviate this issue, since it will block CD59’s impact on MAC enhancing CDC and help overcome CDC resistance of tumors expressing CD59 (115, 144). Similarly, complement inhibition is being explored as a potential strategy to mitigate side effects associated with CAR T cell therapies, such as cytokine release syndrome with the full impact of complement inhibition on CAR T cell therapy efficacy and safety still being investigated (145, 146). Further, the intracellular complement system (complosome) plays a role in regulating T cell responses (147), but systemic complement inhibition may not affect this intracellular activity. Future studies may provide more definitive insights into the relationship between complement inhibition and CAR T cell therapy outcomes.

### *Systemic manipulation of complement system*.

Systemic complement modulation offers therapeutic opportunities by targeting complement proteins in various pathways. Inhibiting early components of the classical or lectin pathways, such as C1q, C2, and MASP2, could prevent disease-promoting actions while preserving cascade functionality and its many downstream effectors (148). Blocking the terminal pathway at C5 can counteract proinflammatory C5a signaling and MAC-induced cell death. Selective blockade of the C5a/C5aR1 axis can inhibit C5aR1 signaling while preserving the effects of C5aR2 and the formation of MAC on opsonized surfaces (149). On the other hand, targeting C3 protein by inhibiting its activity can have broader effects than inhibiting C5. Inhibiting C3 by using the C3 blocker, Cp40 analog, will impede the production of subsequent effector molecules and affect the functions of immunomodulatory and effector B and T cells (150). The advantage of small-molecule inhibitors is that all of them are orally administered and offer the possibility of enhancing patient compliance for chronic illnesses, in contrast with biologics, which are given by intravenous or subcutaneous routes. Nevertheless, it is crucial to use prudence while administering the dosage, as even minute quantities of the enzyme can suffice to trigger the pathways.

### *Local manipulation of the complement system*.

Local complement modulation has shown promise in treating diseases with localized pathology. For example, phase III trials demonstrated the effectiveness of pegcetacoplan (Syfovre), a C3 inhibitor, and avacincaptad pegol (Zimura), a C5-targeting RNA aptamer, in geographic atrophy, an advanced state of macular degeneration (151). Both drugs use polyethylene glycol linkers, which could pose risks of tissue accumulation over time. Gene therapy platforms based on adeno-associated viral vectors offer targeted, less frequent dosing, and improved control over complement activation by regulating the expression of complement modulators such as CR2-FH and CR2-Crry (152). Mini-FH (SCR1–4 and SCR19–20 of FH), a fusion construct mimicking FH, has shown therapeutic effectiveness superior to the full-length protein in ex vivo models such as paroxysmal nocturnal hemoglobinuria (PNH) and periodontitis models (153). The effectiveness of mini-FH appears to vary depending on the specific disease model and the mechanisms involved, and its impact on RCC still remains to be investigated.

### *Complement targeting in RCC*.

Research into complement-targeted therapies for RCC is still in its infancy. As mentioned earlier, RCCs survive by expressing complement-regulatory proteins that protect them from complement-mediated lysis, grow and metastasize by promoting angiogenesis (75) through C3a and C5a and by suppressing antitumor immunity recruiting immune cells, such as Tregs or MDSCs. Complement therapies could include both direct modulation of complement proteins or in combination with ICIs. In a murine model resistant to ICI, inhibitors of C3aR1 (SB290157) and C5aR1 (PMX53) reduced tumor growth and TIL function, increasing IFN-γ production (100). Direct complement therapies can include C5aR antagonists, C3 inhibitors, blocking complement regulatory proteins, and modulating cytotoxicity by targeting complement pathways such as with anti-C5 antibodies. Combining complement inhibition with immunotherapy could enhance antitumor immune responses, such as by (a) inhibiting the complement-induced recruitment of Tregs and MDSCs; (b) increasing the effectiveness of ICIs, which work by reactivating T cells to attack the tumor; (b) enhancing the activity of DCs and macrophages, thereby promoting antigen presentation to T cells and generating a stronger and sustained immune response; (c) reducing immune suppression and promoting the infiltration of effector immune cells that are activated by checkpoint inhibitors; and (d) using antiangiogenic therapies (e.g., VEGF inhibitors, such as sunitinib or pazopanib) to inhibit the formation of blood vessels, limiting tumor growth and metastasis. Results from a study evaluating the efficacy of (100) nivolumab alone or in combination with ipilimumab in RCC patients (154) showed that patients with lower levels of FH and complement factor D (FD) had a less favorable response to ICIs, whereas patients with reduced levels of complement factor I (FI) and TCC were associated with better responses and prolonged periods without tumor progression. Combining anti-C5a and anti–PD-1 monoclonal antibodies significantly reduced tumor growth in a lung adenocarcinoma mouse model, indicating potential synergy between complement inhibition and ICI therapy (101). However, there are challenges such as balancing complement activation and suppression, since complement has both protumor and antitumor effects, depending on the context. Different renal cancers subtypes and patient-specific factors (e.g., genetic mutations, preexisting immune profiles) may impact how complement-targeting therapies interact with the TME and therefore personalize the approaches. And finally, since complement inhibition can have systemic effects, monitoring and management will be necessary to prevent impairment of the body’s ability to fight infections. Although these findings are preliminary, they are promising and highlight the need for further research to explore the therapeutic potential of complement modulation alone or in combination with immunotherapy.

### *Clinical trials*.

Several clinical trials are currently exploring complement inhibition in cancer therapy. Clinical trials to evaluate the complement-targeted therapies include using C3 and C5aR1 as specific targets, complement inhibitors and ICIs (e.g., anti–PD-1 or anti–CTLA-4 therapies), and antiangiogenic agents with complement-targeting strategies. A phase 1 clinical trial (STELLAR-001, ClinicalTrials.gov NCT03665129 by Innate Pharma and MedImmune) assessed the effectiveness of IPH5401, a monoclonal C5aR antibody, in combination with anti–PD-L1 therapy, durvalumab, in patients with advanced solid tumors (155). It was halted due to a lack of significant improvement in clinical outcomes, such as progression-free survival (PFS) or overall survival (OS), compared with existing treatment regimens. Complement inhibition can be a delicate balancing act, as the complement system also plays critical roles in immune activation and tumor cell killing, and the dose and timing of complement inhibition may not have been optimal for achieving the desired therapeutic effect, reducing the efficacy of the treatment, leading to a lack of clinical benefit. While the STELLAR trial’s outcome is disappointing, it does not necessarily signal the end of complement inhibition as a therapeutic approach for solid tumors. A second trial (results are pending) is assessing the effectiveness of the anti-C5aR monoclonal antibody TJ210001 as a standalone treatment (I-Mab Biopharma, NCT04678921). The clinical testing of C3 extracellular inhibition (Apellis Pharmaceuticals, pegcetacoplan) is currently underway in ovarian malignancies, either in combination with pembrolizumab (anti–PD-1) or with a combination of bevacizumab (anti-VEGF) and pembrolizumab (156). Anti–PD-1 and anti-VEGF treatments can cause complement activation that generates C5a, which can increase tumor growth by recruiting MDSCs and reducing the antitumor properties of T cells. Therefore, it is expected that the combination therapy will suppress complement activation and improve the antitumor efficacy of these therapeutics (41, 157, 158). The results of these studies will provide valuable insights for future complement-based cancer therapies. Given the role of intracellular complement in immune cells, developing cancer-specific intracellular targeting approaches might be necessary for optimal effectiveness. In addition, the expanding and diverse gene therapy toolbox (159, 160) that includes gene augmentation, editing of RNA, base, prime, and CRISPR/Cas9 remains untapped and provides hope for therapy where traditional therapeutics fail.

## Conclusion

A deeper understanding of the complement system’s involvement in kidney cancer has the potential to lead to the development of biomarkers for prognosis and therapy. This can aid in the design of novel treatments targeting specific complement pathways, enhancing the efficacy of existing treatments or overcoming resistance mechanisms. Furthermore, insights into complement-mediated mechanisms in kidney cancer could pave the way for personalized medicine approaches to tailor treatments to individual patients based on their complement profiles.

In conclusion, complement therapeutics represents a rapidly evolving field in kidney cancer treatment. By targeting the interactions between complement pathways and tumor biology, we can deepen our understanding of the disease pathogenesis and explore new avenues for improving patient outcomes. As clinical trials advance and our knowledge of the complement system deepens, integrating complement-based strategies into clinical practice could profoundly transform kidney cancer management, offering hope for improved outcomes and new treatment options for patients.

## Version 1 05/01/2025

Electronic publication
